# [Corrigendum] Spicatoside A in red *Liriope platyphylla* displays a laxative effect in a constipation rat model via regulating mAChRs and ER stress signaling

**DOI:** 10.3892/ijmm.2024.5462

**Published:** 2024-11-25

**Authors:** Ji Eun Kim, Jun Go, Hee Seob Lee, Jin Tae Hong, Dae Youn Hwang

Int J Mol Med 43: 185-198, 2019; DOI: 10.3892/ijmm.2018.3960

Following the publication of the above article, an interested reader drew to the Editor's attention that various of the histological structural images shown in Fig. 5 on p. 190 were strikingly similar to data that were featured in Fig. 1A of a previous paper by the same research group that appeared in the Journal *Laboratory Animal Research*. On re-examining their original data, the authors confirmed that an error occurred during the paper submission/production process, and that Fig. 5 did not appear in the above article as the authors had intended.

The correct version of Fig. 5, containing the data that the authors intended for inclusion in this article, is shown on the next page. Also shown is a corrected version of Table II corresponding to the replacement version of Fig. 5, containing data that are derived from an analysis of the data shown in this figure. Furthermore, the replacement of the images in Fig. 5, and the revisions of the data made in Table II, also dictate that the following changes are needed to be made in the main text of the paper (all associated with Results section on p. 191, '*Recovery effect of EtRLP on histological alterations of the transverse colon*' subsection): The sentences in lines 9-14 of this section should now read as following (changes from the original text are highlighted in **bold**): 'Following Lop+EtRLP or Lop+Bisac treatments, the villus length increased by **270-290%** relative to the Lop+Vehicle-treated group (Fig. 5 and Table II). Furthermore, the alterations in **muscle thickness** were similar to those in villus length, although crypt layer thickness only increased by **145-150%** relative to the **Lop+Vehicle-treated group** (Fig. 5 and Table II)'.

Note that the errors made during the assembly of Fig. 5 and Table II did not grossly affect the overall conclusions reported in the paper. All the authors agree with the publication of this corrigendum, and are grateful to the Editor of *International Journal of Molecular Medicine* for allowing them the opportunity to publish this. They also apologize to the readership for any inconvenience caused.

## Figures and Tables

**Figure 5:**
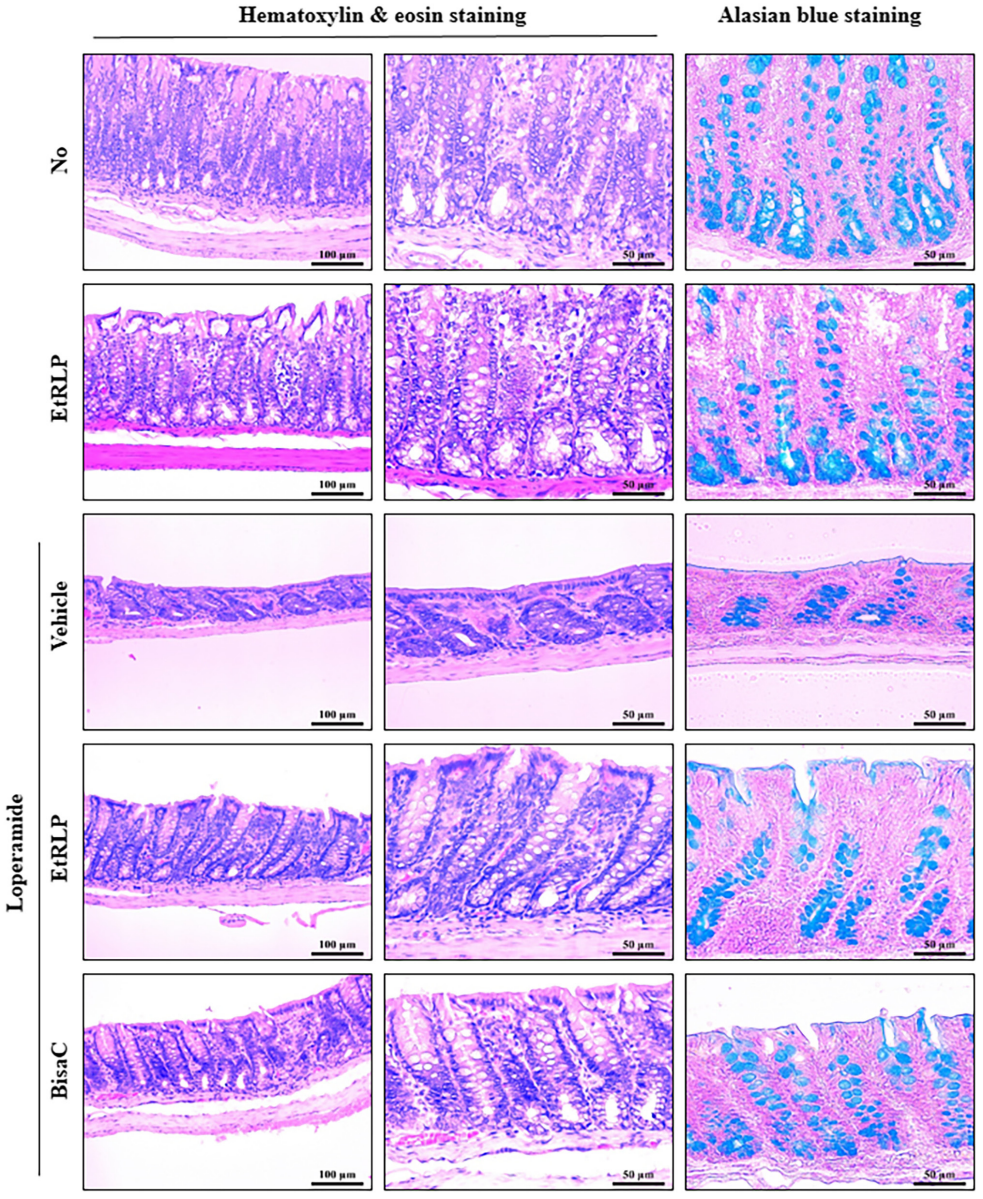
Histological structure of transverse colon collected from Lop-induced constipated rats. Following the collection of transverse colons from the Non-, EtRLP-, Lop+Vehicle-, Lop+EtRLP- and Lop+BisaC-treated groups, H&E stained sections of these tissues were observed at two magnifications via light microscopy (left and middle column). Mucin on the tissue sections was stained with alcian blue (right column). Crypt layer thickness, muscle thickness, and villus length in transverse colon were measured in H&E stained images with the Leica Application Suite software. EtRLP, Red Liriope platyphylla extract; Lop, loperamide; BisaC, bisacodyl; H&E, hematoxylin and eosin.

**Table II:** Histological parameters of experimental rats with Lop-induced constipation.

Categories	Non-treated E	tRLP	Loperamide		
			Vehicle	EtRLP	BisaC
Villus length (*μ*m)	287.9±14.0	228.7±20.1	79.5±14.1^a^	225.5±14.6^b^	217.0±15.0^b^
Muscle thickness (*μ*m)	70.6±4.0	63.0±4.3	22.4±1.5^a^	55.2±3.6^b^	55.4±4.9^b^
Crypt layer thickness (*μ*m)	111.1±37.0	101.5±26.1	51.2±2.2^a^	76.6±14.7^b^	75.3±16.2^b^
Number of goblet cell (ea)	315.4±16.3	316.4±18.7	161.3±8.1^a^	256.0±17.7^b^	256.8±20.6^b^
Number of crypt of lieberkuhn (ea)	30.2±3.3	25.8±3.3	11.8±3.5^a^	19.8±2.9^b^	19.6±3.8^b^

a P<0.05 compared with the non-treated group;

b P<0.05 compared with the Lop+Vehicle-treated group. Lop, loperamide; EtRLP, Red *Liriope platyphylla* extract; BisaC, bisacodyl.

